# Genetic Ablation of *Fgf23* or *Klotho* Does not Modulate Experimental Heart Hypertrophy Induced by Pressure Overload

**DOI:** 10.1038/s41598-017-10140-4

**Published:** 2017-09-12

**Authors:** Svetlana Slavic, Kristopher Ford, Magalie Modert, Amarela Becirovic, Stephan Handschuh, Andreas Baierl, Nejla Katica, Ute Zeitz, Reinhold G. Erben, Olena Andrukhova

**Affiliations:** 1 Department of Biomedical Sciences, University of Veterinary Medicine Vienna, Vienna, Austria; 2 VetCore, University of Veterinary Medicine Vienna, Vienna, Austria; 3 Department of Statistics and Operations Research, University of Vienna, Vienna, Austria

## Abstract

Left ventricular hypertrophy (LVH) ultimately leads to heart failure in conditions of increased cardiac pre- or afterload. The bone-derived phosphaturic and sodium-conserving hormone fibroblast growth factor-23 (FGF23) and its co-receptor Klotho have been implicated in the development of uremic LVH. Using transverse aortic constriction (TAC) in gene-targeted mouse models, we examine the role of Fgf23 and Klotho in cardiac hypertrophy and dysfunction induced by pressure overload. TAC profoundly increases serum intact Fgf23 due to increased cardiac and bony *Fgf23* transcription and downregulation of Fgf23 cleavage. Aldosterone receptor blocker spironolactone normalizes serum intact Fgf23 levels after TAC by reducing bony *Fgf23* transcription. Notably, genetic *Fgf23* or *Klotho* deficiency does not influence TAC-induced hypertrophic remodelling, LV functional impairment, or LV fibrosis. Despite the profound, aldosterone-mediated increase in circulating intact Fgf23 after TAC, our data do not support an essential role of Fgf23 or Klotho in the pathophysiology of pressure overload-induced cardiac hypertrophy.

## Introduction

Left ventricular hypertrophy (LVH) occurs together with reactive interstitial and perivascular fibrosis as an adaptive response aimed to reduce wall stress during increased pre- or afterload. However, prolonged increases in workload lead to cardiomyocyte death, replacement fibrosis, and transition to heart failure (HF)^1^. Both LVH and cardiac fibrosis are strong and independent predictors of cardiovascular mortality^2, 3^. To reduce the incidence of HF in risk patients, a better understanding of the mechanisms underlying the pathophysiology of hypertrophy is needed. In this context, augmented fibroblast growth factor-23 (FGF23) signalling as well as Klotho deficiency have been implicated as novel mediators of heart hypertrophy in the general population and in patients with chronic kidney disease (CKD)^4–6^.

FGF23 is a phosphaturic, Na^+^ and Ca^2+^-preserving hormone with an important regulatory function on 1,25(OH)_2_D_3_ metabolism^7–9^. FGF23 is normally mainly synthesized by osteocytes and osteoblasts, but under certain pathological conditions its synthesis can be increased also in extra-osseous tissues, such as heart, spleen, calcified coronary arteries, and kidney^10–14^. At physiological concentrations, binding of FGF23 to FGF receptors (FGFR) on target cells is mediated by αKlotho (further Klotho), a transmembrane protein which associates with FGFRs to increase their binding affinity for FGF23^15, 16^. Klotho also exists in a soluble form, which is generated by shedding of transmembrane Klotho or by alternative splicing of the *Klotho* gene^17, 18^. The co-receptor function of Klotho is essential for the renal actions of FGF23^15^, but the role of Klotho in the cardiovascular system is controversial^19^. In the heart, expression of Klotho is limited to the sinoatrial node^20^. Although a protective role of soluble Klotho in the development of LVH has been reported in animal models^21^, clinical studies failed to confirm an association between soluble Klotho and cardiovascular mortality in patients with normal renal function^22^. Elevated serum FGF23 levels were found to be strongly associated with LVH in patients with reduced kidney function^4, 5^. On the other hand, cardiac dysfunction and hypertrophy are not universal findings in patients with FGF23-related hypophosphatemic rickets characterised by chronically elevated FGF23^23, 24^. Therefore, the role of FGF23 and Klotho in the pathophysiology of heart hypertrophy is still unclear.

*In vitro*, FGF23 induced cardiomyocyte (CM) hypertrophy by Klotho-independent binding to FGFR4, activating the pro-hypertrophic phospholipase Cγ/calcineurin/nuclear target of activated T cells (NFAT) signalling pathway^25^. *In vivo* experimental evidence of FGF23-mediated LVH is mainly based on studies performed in experimental CKD models. Driven by deterioration of renal function, FGF23 serum levels rise progressively in CKD. However, the pathophysiology of CKD is complex, and characterized by the presence of additional factors such as electrolyte disturbance, hypervolemia and hypertension. We recently reported that FGF23 increases renal calcium and sodium reabsorption^7, 8^, which may favour vessel calcifications, leading to increased afterload, and may increase preload due to renal sodium preservation and subsequent hypervolemia. Thus, FGF23-mediated hypertrophy in CKD is probably multifactorial rather than solely caused by a direct pro-hypertrophic action on cardiomyocytes.

Due to the early lethality of *Fgf23* deficient mice, a direct proof that FGF23 plays an essential role in mediating cardiac hypertrophy is missing. *Fgf23* ^−/−^ and *Klotho* ^−/−^ mice suffer from intoxication with 1,25(OH)_2_D_3_ due to the lack of FGF23-mediated inhibition of renal 1α-hydroxylase, resulting in hypercalcemia and hyperphosphatemia, which are responsible for the premature aging-like phenotype and the early mortality^9, 26^. To overcome this, we generated *Fgf23* ^−/−^ */*VDR^Δ/Δ^ and *Klotho* ^−/−^/VDR^Δ/Δ^ compound mutants by crossing *Fgf23* ^−/−^ and *Klotho* ^−/−^ mice with mice expressing a non-functioning vitamin D receptor (VDR^Δ/Δ^)^26–28^. To normalize calcium and phosphate homeostasis in VDR^Δ/Δ^ mice, all mice were maintained on a so-called rescue diet, enriched with calcium, phosphorous, and lactose. Transverse aortic constriction (TAC) is the most commonly used model of pressure overload-induced cardiac hypertrophy^29^. To test the hypothesis that Fgf23 and Klotho play an essential role in the development of cardiac hypertrophy induced by increased afterload, we induced TAC in wild-type (WT), VDR^Δ/Δ^, *Fgf23* ^−/−^/VDR^Δ/Δ^, and *Klotho* ^−/−^/VDR^Δ/Δ^ mice. In addition, we investigated the possible mechanisms of the TAC-induced upregulation of cardiac and bony Fgf23 production. We found that neither *Fgf23* nor *Klotho* deficiency modulates cardiac hypertrophy induced by pressure overload.

## Results

### Increased cardiac afterload up-regulates Fgf23 expression

**Figure 1:**
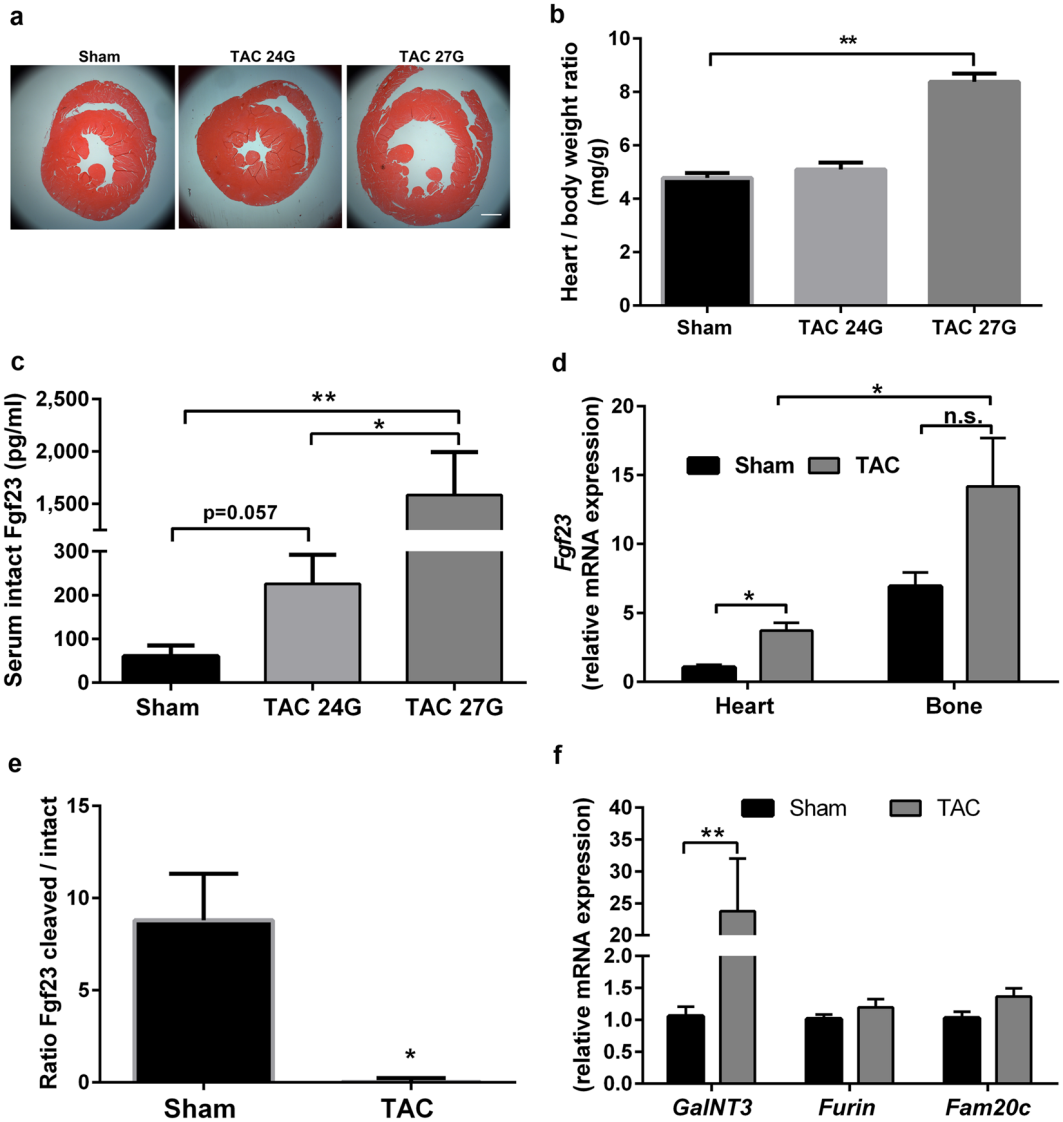
Pressure overload by transverse aortic constriction (TAC) up-regulates Fgf23 expression in WT mice. (**a**) Representative H&E-stained cardiac cross-sections, 4-weeks after sham or TAC surgery (scale bar: 1000 µm). (**b**) Heart/body weight ratio is significantly increased after TAC when constriction was performed with a 27G-needle (n = 6–7). (**c**) Serum intact Fgf23 levels (n = 4–5). (**d**) mRNA expression of *Fgf23* in the heart (left ventricle and septum) and bone (lumbar vertebra L5) (n = 5–6), normalised to expression of *ornithine decarboxylase antizyme (OAZ)*. (**e**) Cleaved serum Fgf23 calculated as C-terminal Fgf23 - intact Fgf23 and presented as ratio of cleaved Fgf23/intact Fgf23 (n = 6–8). (**f**) Cardiac mRNA expression of genes involved in Fgf23 processing. *GalNt3: N-Acetylgalactosaminyl-transferase 3, Furin and Fam20C* (n = 5–7). Data were obtained 4-week post-surgery. Values are mean ± SEM. *p < 0.05, **p < 0.01. If not otherwise specified, TAC was performed using a 27 G needle.

We previously reported increased serum intact Fgf23 levels in experimental myocardial infarction models^10^. Therefore, we first examined whether increased afterload also affects circulating Fgf23 levels. TAC was performed in WT animals using two different pressure grades by ligating the aorta to the lumen of a 24-gauge (OD 0.57 mm) or a 27-gauge (OD 0.41 mm) needle. Left ventricular (LV) fractional shortening (FS) decreased (Suppl. Figure 1a), while heart/body weight ratio and lung oedema increased in parallel with the increased afterload in TAC animals compared to sham, 4 weeks post-surgery (Fig. 1a,b and Suppl. Figure 1b respectively). Interestingly, serum intact Fgf23 levels were also elevated in an afterload dependant manner (Fig. 1c). Using a time course study, we found a profound up-regulation of circulating intact Fgf23 levels within 24 hours after TAC, which was sustained for at least 4 weeks post-surgery (Suppl. Figure 1c).

To define the site of increased Fgf23 production, we analysed transcript levels of *Fgf23* mRNA in the heart and in bone, 4 weeks post-surgery. TAC significantly increased cardiac *Fgf23* gene expression (Fig. 1d). Albeit statistically non-significant, a tendency for increased *Fgf23* expression was also observed in bone of TAC animals, relative to sham. Overall, bone exhibited significantly higher transcript levels of *Fgf23* after TAC compared to the heart, suggesting that upregulated bony production may largely account for the increased circulating Fgf23 levels during pressure overload. In an attempt to further explore the discrepancy between highly elevated serum intact Fgf23 and only moderate induction of cardiac *Fgf23* expression after TAC, we analysed molecules known to be important for the regulation of Fgf23 cleavage. Indeed, pressure overload dramatically reduced the occurrence of cleaved Fgf23 in serum (Fig. 1e). Consistent with a decreased cleavage of Fgf23, we found significantly increased cardiac expression of *GalNT3* (N-Acetylgalactosaminyl-transferase 3), an enzyme O-glycosylating and thereby stabilising FGF23^30, 31^, 4 weeks post-TAC (Fig. 1f). In contrast, bony expression of *GalNT3* was not changed after TAC (data not shown). Cardiac expression of *Fam20c* and *Furin*, both promoting Fgf23 cleavage, remained unchanged after TAC (Fig. 1f). Collectively, these data suggest that reduced Fgf23 cleavage may contribute to increased intact Fgf23 concentrations in the heart during pressure overload stress.

Next, we analysed serum biochemistry to test whether mineral ion alterations were associated with the increased serum Fgf23 levels induced by pressure overload. However, serum levels of phosphate, calcium, sodium, potassium, and iron were not significantly changed as compared to sham animals, 4 weeks after TAC. This excludes the possibility that increased phosphate and/or reduced iron levels are driving Fgf23 secretion following TAC. Mild, but significant increases in serum creatinine, urea and alkaline phosphatase levels were noted in TAC mice, 4 weeks post-surgery (Suppl. Table 1.).

### The pressure overload-induced increase in serum Fgf23 levels is mediated by aldosterone

**Figure 2:**
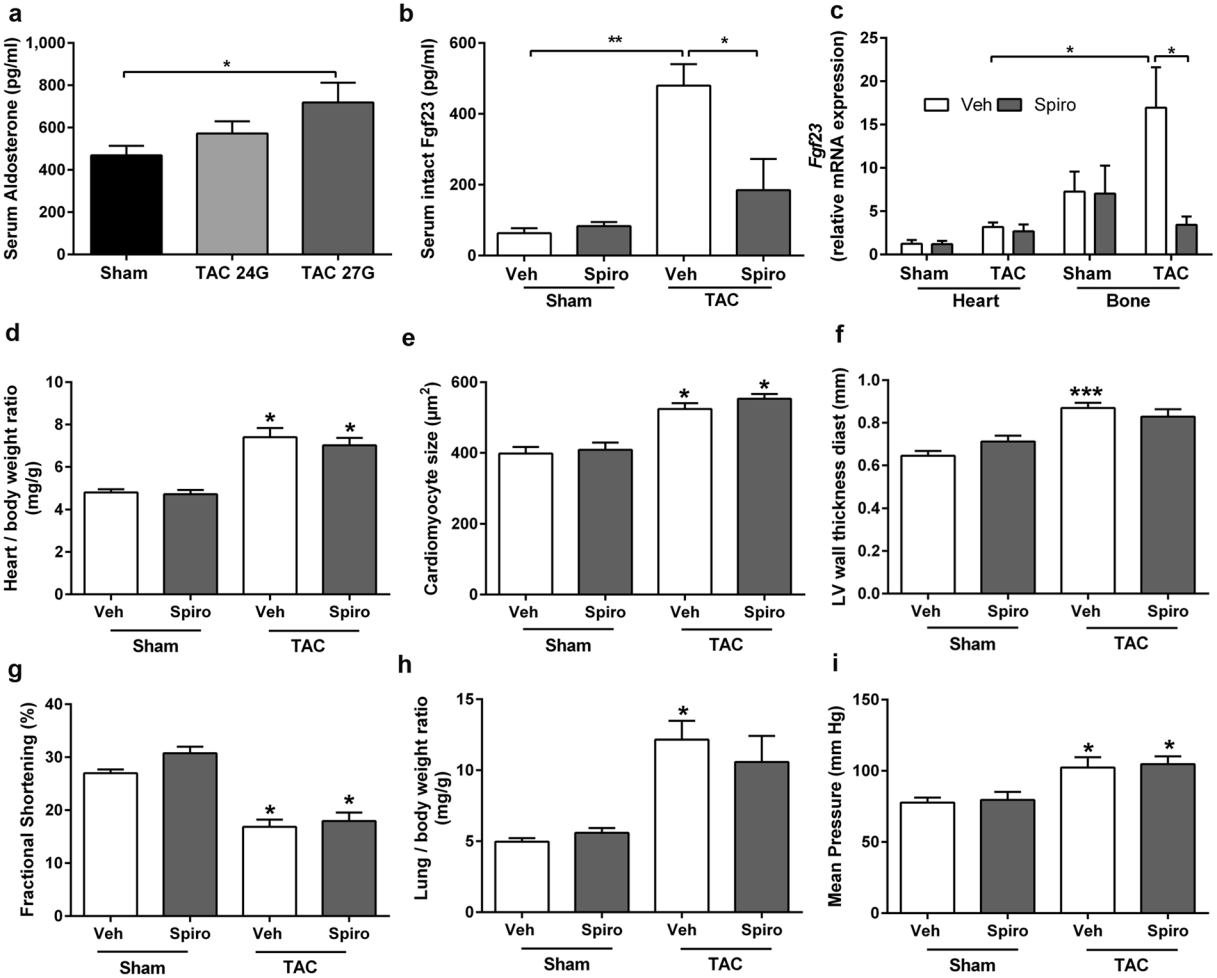
Effect of spironolactone treatment on FGF23 levels, morphological and functional parameters, 2 weeks after TAC. (**a**) Serum aldosterone levels 4-week post-surgery (n = 4–5). (**b**) Spironolactone (Spiro) effect on serum intact Fgf23 levels after TAC (n = 4–6). (**c**) Effect of spironolactone treatment on cardiac and bony *Fgf23* mRNA expression (lumbar vertebra L5) after TAC (n = 3–7). (**d**–**f**) Spironolactone did not affect cardiac hypertrophy development measured as (**d**) heart/body weight ratio, (**e**) mean cross-sectional area of cardiomyocytes and (**f**) thickness of the left ventricular (LV) wall measured by echocardiography. (**g**) Reduced cardiac function after TAC measured as fractional shortening by echocardiography is not affected by spironolactone treatment (**h**) Spironolactone treatment does not prevent development of lung oedema in TAC mice. (**i**) Effect of spironolactone (Spiro) treatment on mean blood pressure in Sham and TAC, measured proximal to the ligation by intra-aortic pressure catheter (n = 4–6). Data in (**b**–**i**) were obtained after 2 weeks of daily gavage with vehicle (Veh) or spironolactone (Spiro). n = 4–7 if not otherwise specified; mean ± SEM, in **a–c** *p < 0.05, **p < 0.01; in **d–i** *p < 0.05 and ***p < 0.001 vs. sham control of the same treatment regimen.

Since aldosterone was shown to stimulate FGF23 expression *in vitro* ^32^, we next examined serum aldosterone levels in sham and TAC mice. Indeed, serum aldosterone levels were significantly increased in TAC animals, and followed a similar afterload-dependent pattern, compared with circulating intact Fgf23 levels (Fig. 2a). To investigate the role of aldosterone in the upregulation of Fgf23 secretion after TAC, we orally treated WT animals with the mineralocorticoid receptor inhibitor spironolactone over 2 weeks post-TAC. Spironolactone treatment increased urinary sodium excretion in TAC mice, whereas urinary potassium was not significantly changed (Suppl. Figure 2a,b). Serum sodium and potassium concentrations remained unchanged (data not shown). Renal function as measured by glomerular filtration rate was similarly reduced in all TAC animals, independent of spironolactone treatment (Suppl. Figure 2c). The administered dosage of spironolactone did not alter mean aortic pressure (Fig. 2i). Interestingly, spironolactone suppressed the rise in serum Fgf23 levels following TAC, but had no influence on basal Fgf23 levels in Sham mice (Fig. 2b). To define the target organ of the suppressive effect of spironolactone treatment on circulating intact Fgf23 levels in TAC mice, we analysed *Fgf23* mRNA abundance in heart and bone. Spironolactone treatment did not influence transcription of *Fgf23* in the heart, but significantly reduced transcription of *Fgf23* in bone, 2 weeks after TAC (Fig. 2c). Taken together, these data suggest that the pressure overload-induced increase in bony *Fgf23* expression is mainly driven by increased aldosterone secretion in TAC mice. To our surprise, we did not observe a rescue of heart hypertrophy, 2 weeks after spironolactone treatment, despite reduced serum Fgf23 levels (Fig. 2d–f). Parameters of cardiac hypertrophy such as increased heart/body weight ratio, increased cardiomyocyte size and LV wall thickness, reduced fractional shortening, and lung oedema remained unchanged after spironolactone treatment (Fig. 2d–h and Suppl. Figure 2d,e), questioning the importance of increased systemic Fgf23 for the development of heart hypertrophy induced by pressure overload.

### Genetic deletion of Fgf23 or Klotho does not modulate afterload-induced cardiac hypertrophy and pro-hypertrophic signalling

To address the question whether local, paracrine Fgf23 signalling may be involved in the hypertrophic changes during pressure overload, we used a model of genetic deletion of *Fgf23* in the TAC model. In addition, since Klotho was previously shown to protect against uraemia-induced heart hypertrophy^21^, we investigated whether FGF23-independent Klotho signalling may be involved in the pathophysiology of TAC-induced LV hypertrophy. Because genetic disruption of vitamin D signalling rescues the lethal phenotype of *Fgf23* and *Klotho* null mice^26, 27^, we used *Fgf23* ^−/−^/VDR^Δ/Δ^ and *Klotho* ^−/−^/VDR^Δ/Δ^ compound mutants to assess the role of Fgf23 and Klotho in the pathophysiology of cardiac hypertrophy. Mice with a non-functioning VDR (VDR^Δ/Δ^) were used as controls.

**Figure 3:**
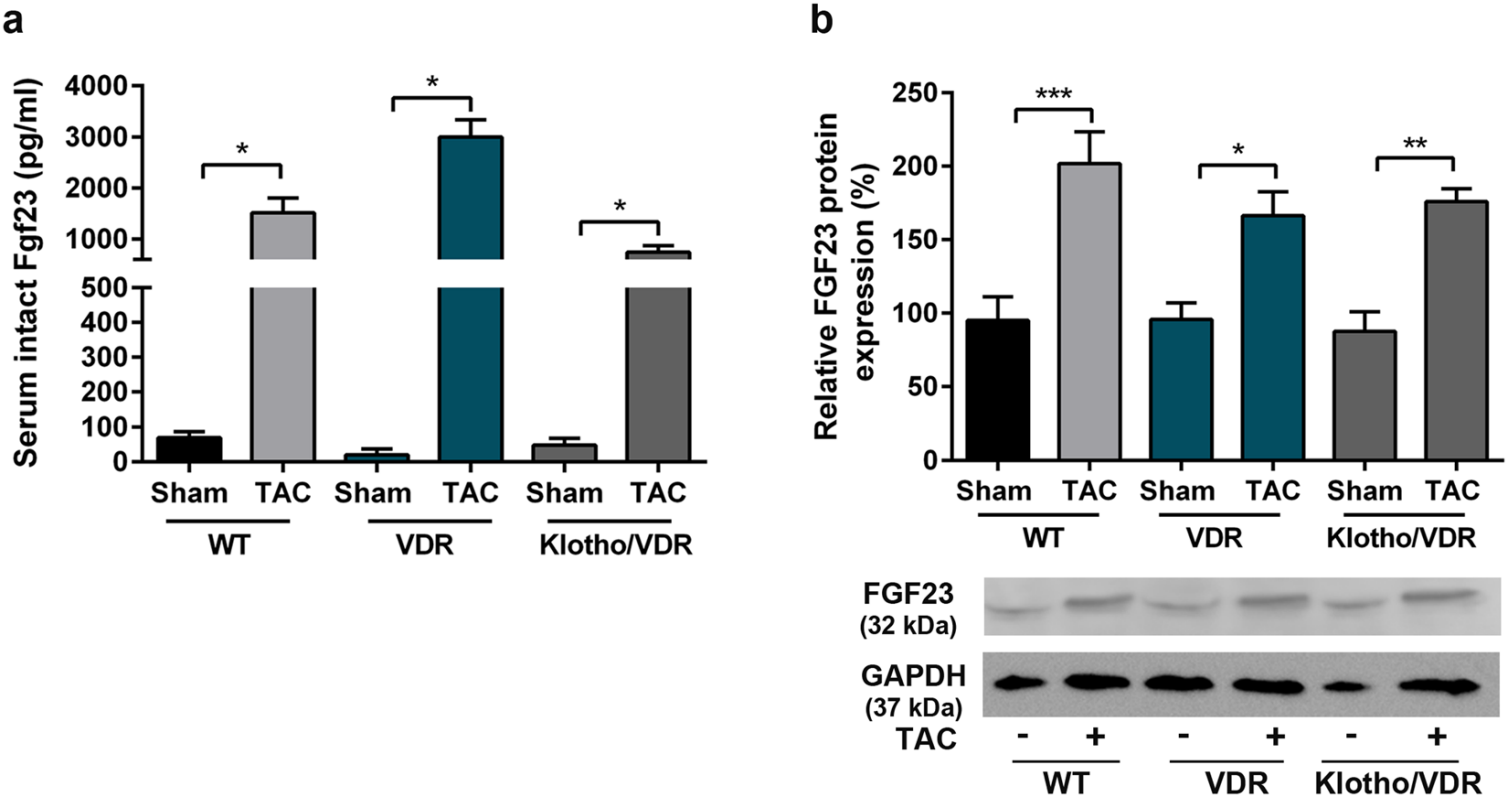
VDR deficiency does not alter the TAC-induced increase in circulating Fgf23 and in cardiac Fgf23 protein expression. (**a**) Serum intact Fgf23 levels measured by ELISA (n = 4–7), and (**b**) Western blot analysis of Fgf23 protein expression in the left ventricle normalised to GAPDH expression (n = 3) in WT, VDR^Δ/Δ^ and *Klotho* ^−/−^/VDR^Δ/Δ^ mice, 4 weeks after TAC surgery. Data are mean ± SEM, *p value < 0.05, **p < 0.01, ***p < 0.001.

To address the question whether a VDR deficient background influences the response of mice to TAC, we first assessed the TAC-induced increase in serum and cardiac levels of Fgf23 in the different genotypes. Circulating intact Fgf23 and cardiac Fgf23 protein expression did not differ in sham WT, VDR^Δ/Δ^ and *Klotho* ^−/−^/VDR^Δ/Δ^ mice on rescue diet, and were profoundly increased after TAC in all genotypes to levels beyond 1,000 pg/mL (Fig. 3a and b). We did not directly compare serum intact Fgf23 levels in the different TAC groups, because the different genotypes were analysed in separate immunoassays. Fgf23 was not detectable in serum of *Fgf23* ^−/−^/VDR^Δ/Δ^ mice by ELISA (data not shown). Importantly, there was no difference in the hypertrophic response of WT and VDR deficient mice to TAC (Suppl. Figure 3a–c). Moreover, a 5-day i.p. treatment with recombinant FGF23 (rFGF23) induced a similar degree of LV hypertrophy and a similar increase in mean arterial pressure in WT and VDR mutant mice (Suppl. Figure 3d–f). Taken together, these data demonstrate that a VDR deficient background neither blunts the TAC-induced increase in FGF23 expression nor the prohypertrophic actions of FGF23, and does not modulate the hypertrophic response to TAC.

**Figure 4:**
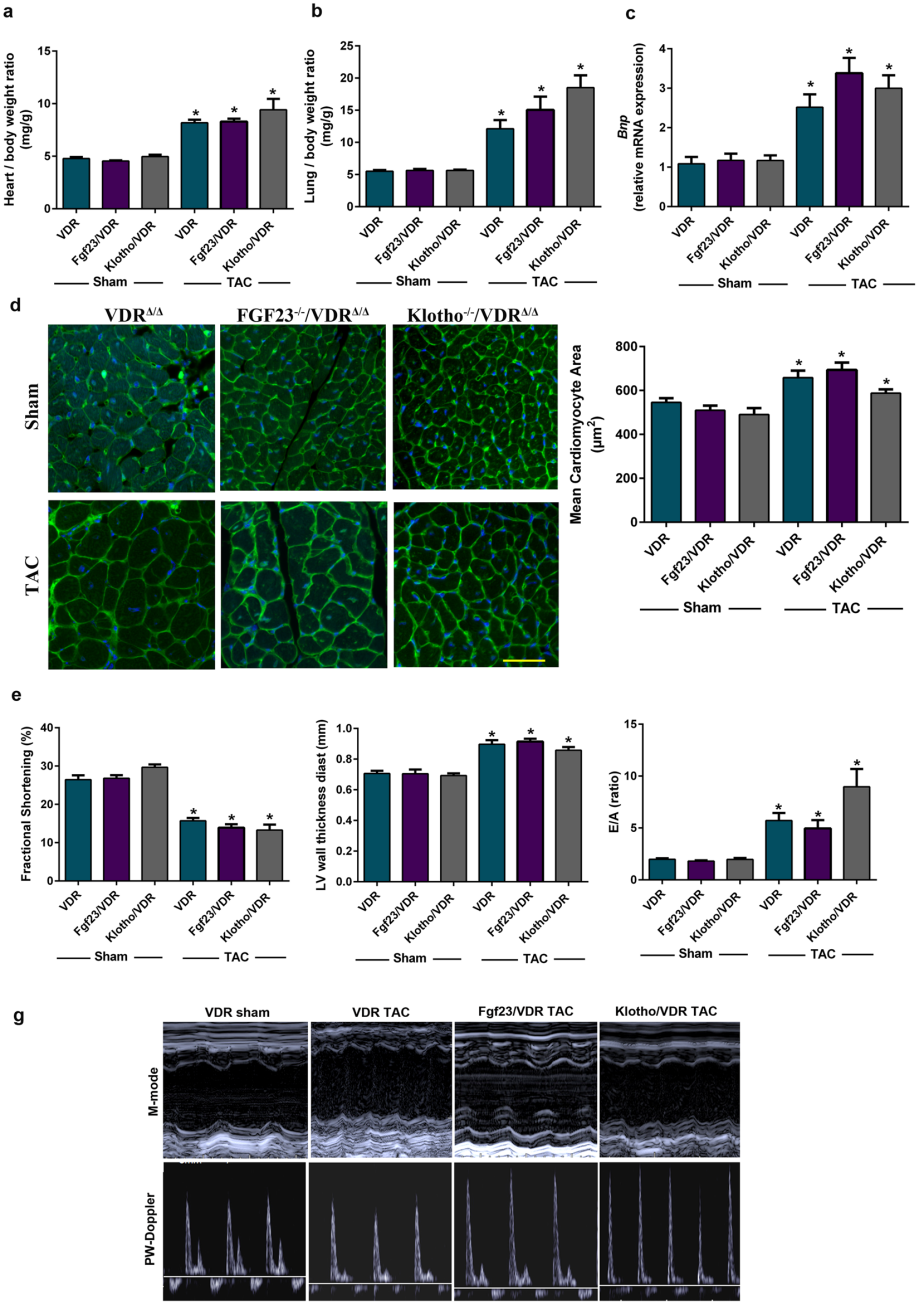
Loss of Fgf23 or Klotho does not modulate afterload-induced cardiac hypertrophy. (**a**) Heart/body weight ratio and (**b**) lung/body weight ratio (n = 6–10). (**c**) Cardiac mRNA expression of brain natriuretic peptide (*Bnp*, n = 5–6). (**d**) Left: Representative FITC-labelled wheat germ agglutinin (WGA)-stained sections (Scale bar: 50 µm). Right: Quantification of mean cardiomyocyte size after FITC-WGA staining (n = 6–9). (**e**) Fractional shortening, left ventricular wall thickness and diastolic functional parameter (E/A ratio) measured by echocardiography 4-weeks post-surgery (n = 6–9). (**g**) Representative echocardiograms of M-mode and pulsed-wave Doppler analysis in sham VDR^Δ/Δ^ and in TAC VDR^Δ/Δ^, *Fgf23* ^−/−^/VDR^Δ/Δ^ and *Klotho* ^−/−^/VDR^Δ/Δ^ mice. Data in (**a**–**e**) presented as mean ± SEM for VDR^Δ/Δ^, *Fgf23* ^−/−^/VDR^Δ/Δ^ and *Klotho* ^−/−^/VDR^Δ/Δ^ mice after sham and TAC (27 G) surgery. *p < 0.05 vs. sham control of the same genotype.

Aortic pressure after TAC in the ascending aorta proximal to the ligation was similar in all groups confirming that the same grade of pressure-overload was induced in all three genotypes (Suppl. Table 2). Survival rate after the TAC procedure was similar in *Fgf23* ^−/−^/VDR^Δ/Δ^ and *Klotho* ^−/−^/VDR^Δ/Δ^ mutant mice compared to VDR^Δ/Δ^ mice (Suppl. Figure 4a). In addition, the TAC-induced changes in heart/body weight ratio, heart weight/femur length ratio, cardiomyocyte size, LV wall thickness, lung oedema, expression of the molecular hypertrophy marker *Bnp*, LV systolic and diastolic function, and LV mass and size were similar in mice of all genotypes (Fig. 4, Suppl. Figure 4, and Suppl. Table 2). Heart rate tended to be higher in TAC mice of all genotypes relative to Sham controls, but this effect reached statistical significance only in VDR mutant mice, but not in *Fgf23* ^−/−^/VDR^Δ/Δ^ and *Klotho* ^−/−^/VDR^Δ/Δ^ mutant mice (Suppl. Figure 4c). Neither did the absence of Fgf23 in *Fgf23* ^−/−^/VDR^Δ/Δ^ animals protect against cardiac hypertrophy, nor did deletion of Klotho lead to further exacerbation of the TAC-induced hypertrophic phenotype, relative to VDR^Δ/Δ^ controls.

These results suggest that factors other than FGF23 are essentially mediating the hypertrophic response during pressure-overload. Previously it was shown that FGF2 signalling is necessary for hypertrophy development during TAC^33^. We also found increased cardiac *Fgf2* mRNA expression in WT mice, 2 weeks (Suppl. Figure 5a) and 4 weeks (not shown) after TAC. In addition, pressure overload increased cardiac *Fgf2* mRNA abundance in VDR^Δ/Δ^, *Fgf23* ^−/−^ */*VDR^Δ/Δ^, and *Klotho* ^−/−^/VDR^Δ/Δ^ mice, 4 weeks post-surgery (Suppl. Figure 5b). Neither spironolactone treatment nor *Fgf23* or *Klotho* deficiency affected the TAC-induced rise in cardiac *Fgf2* expression, which is in accordance with unchanged hypertrophy development in these mice (Suppl. Figure 5, Figs 2 and 4). To test the possible involvement of ERK1/2 signalling in the TAC-induced hypertrophy, we analysed the activation of ERK1/2 after TAC. However, phosphorylation of ERK1/2 in cardiac tissue remained unchanged, 2 weeks after TAC or after spironolactone treatment (Suppl. Figure 5c), indicating that cellular signalling pathways other than ERK1/2 mediate hypertrophy during the early phase of pressure overload. On the contrary, TAC activated cardiac calcineurin/NFAT signalling, as evidenced by reduced expression of pNFAT in histological sections, and by increased transcription of its target gene regulator of calcineurin 1 (rCAN1) (Suppl. Fig. 6). In addition, spironolactone treatment did not affect pNFAT expression in cardiomyocytes of Sham and TAC mice, 2 weeks post-surgery (Suppl. Fig. 6). Similarly, *Fgf23* and *Klotho* deficiency had no influence on cardiac calcineurin/NFAT signalling as shown by similarly increased cardiac rCAN1 gene expression in all TAC animals (Suppl. Fig. 6). Collectively, these data indicate that neither *Fgf23* or *Klotho* deficiency nor spironolactone treatment modulate afterload-induced cardiac pro-hypertrophic signalling.

### Afterload-induced cardiac fibrosis develops independent of Fgf23 and Klotho, and is associated with upregulated cardiac FGF receptor-1 and -3 expression

**Figure 5:**
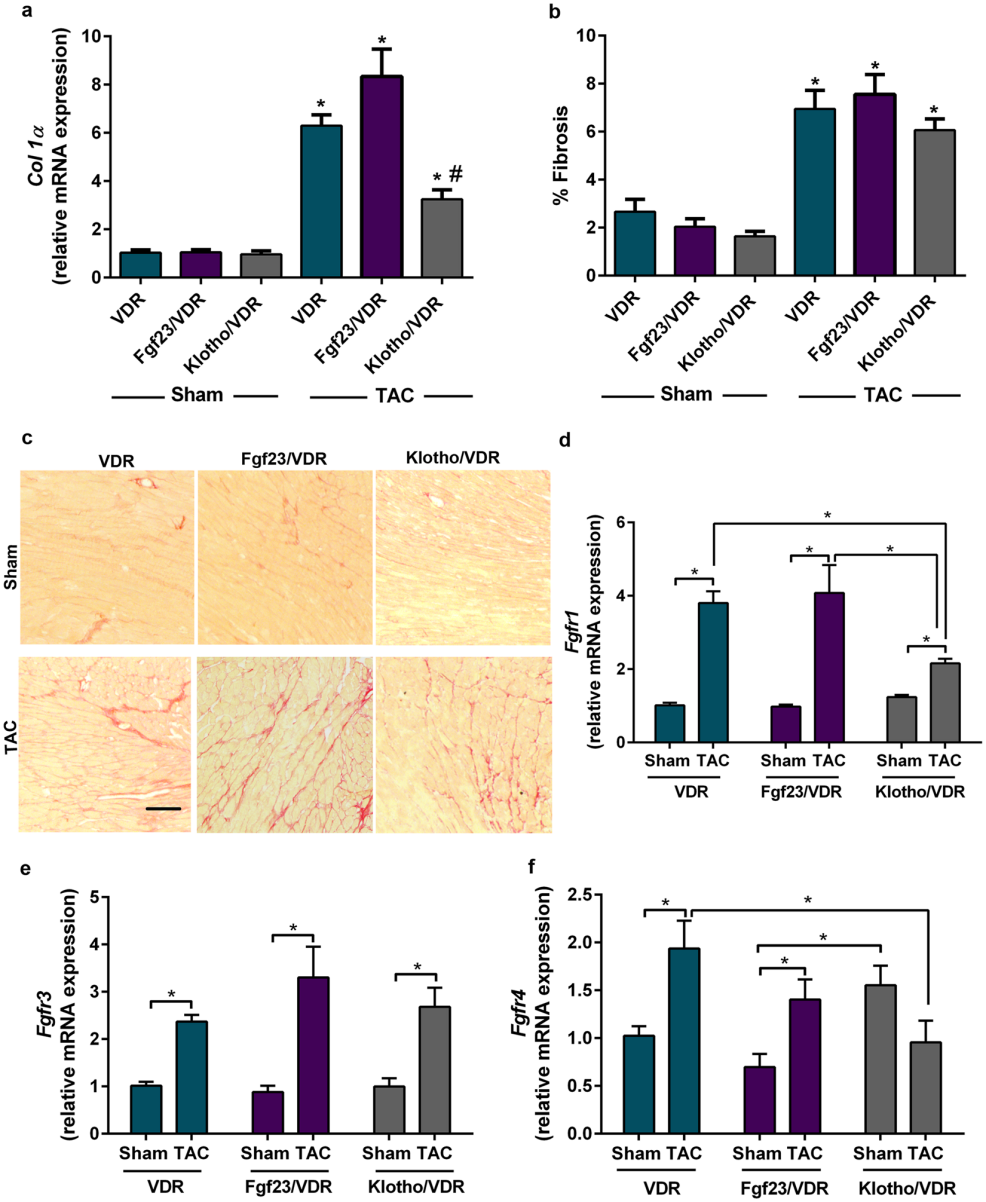
Fgf23 and Klotho deficiency do not protect from afterload-induced cardiac fibrosis. (**a**) Cardiac relative mRNA expression of *Col1α* (n = 5–6). (**b**) Quantification of fibrosis after picrosirius red staining (n = 6–9). (**c**) Representative images of total collagen in cardiac sections after picrosirius red staining (Scale bar: 100 µm). (**d**–**f**) Effect of pressure overload and *Fgf23* or *Klotho* deficiency on cardiac *Fgf* receptor mRNA expression (n = 5–6). Data in (**a**–**f**) are from VDR^Δ/Δ^, *Fgf23* ^−/−^/VDR^Δ/Δ^ and *Klotho* ^−/−^/VDR^Δ/Δ^ mice, 4-weeks after sham and TAC (27 G) surgery. Data are mean ± SEM, in (**a**,**b**) *p < 0.05 vs. sham control of the same genotype; in (**a**) #p < 0.05 vs. VDR^Δ/Δ^ and *Fgf23* ^−/−^/VDR^Δ/Δ^ mice, in (**d**–**f**) *p < 0.05.

Interstitial fibrosis is one of the hallmarks of LV hypertrophic cardiomyopathy. An adverse role of FGF23 in promoting cardiac fibrosis was recently suggested^34^. We found that 4 weeks after TAC, interstitial fibrosis as well as type I collagen (*Col1α*) mRNA expression were increased in the LV and septum in all genotypes (Fig. 5a–c). Interestingly, *Klotho* ^−/−^/VDR^Δ/Δ^ mutants showed reduced cardiac *Col1α* mRNA expression levels compared to VDR^Δ/Δ^ and *Fgf23* ^−/−^/VDR^Δ/Δ^ mutant mice following TAC. Therefore, factors other than Fgf23 seem to be involved in cardiac pro-fibrotic changes upon TAC.

Recently, it was shown in a CKD-mediated cardiac hypertrophy model that FGF23 exerts its pro-hypertrophic action in cardiomyocytes by Klotho-independent signalling through FGFR4^25, 35^. To obtain further insight into potential FGFR-mediated signalling mechanisms in TAC-induced cardiac hypertrophy, we investigated mRNA expression of *Fgfr1*, *Fgfr3* and *Fgfr4* in WT, VDR^Δ/Δ^, *Fgf23* ^−/−^ */*VDR^Δ/Δ^, and *Klotho* ^−/−^/VDR^Δ/Δ^ mice, 4 weeks after TAC. In our experimental settings, cardiac mRNA expression of *Fgfr3* and *Fgfr1* were 100- and 250-fold higher compared to *Fgfr4* mRNA expression in WT Sham mice (Suppl. Fig. 7). TAC consistently increased *Fgfr1* and *Fgfr3* mRNA expression in all investigated genotypes (Fig. 5d,e and Suppl. Fig. 7). In contrast, *Fgfr4* mRNA expression was significantly increased after TAC in *Fgf23* ^−/−^/VDR^Δ/Δ^ and VDR^Δ/Δ^ mice but was unchanged in WT and *Klotho* ^−/−^/VDR^Δ/Δ^ mice (Fig. 5 and Suppl. Fig. 7). The robust TAC-induced upregulation in *Fgfr1* and *Fgfr3* mRNA abundance across all genotypes suggests that augmented FGFR signalling may be an important component of the cardiac response to increased afterload. However, the role of specific FGFRs in this signalling network is only poorly understood.

## Discussion

FGF23 and Klotho have emerged as potential novel players in the pathophysiology of LVH. In the present study, we induced TAC in mice with a genetic deletion of *Fgf23* and *Klotho* to study their role in pressure overload-induced LVH. We demonstrate that TAC profoundly increases serum levels of intact Fgf23, augments cardiac mRNA and protein expression of *Fgf23*, increases cardiac transcription of the Fgf23-stabilizing enzyme *GalNT3*, and increases *Fgf23* transcription in bone by an aldosterone-driven mechanism. Finally, we provide evidence that *Fgf23* and *Klotho* deficiency does not modulate the development of LVH and cardiac dysfunction following TAC.

Clinical evidence suggests Fgf23 as a strong predictor of adverse cardiovascular events in CKD patients^6^. A positive correlation between FGF23 levels and cardiovascular outcomes was also shown in patients with established cardiovascular disease^36, 37^. However, studies in community-based non-CKD populations are less consistent, and the association between circulating FGF23 and major cardiovascular events was attenuated after correction for glomerular filtration rate^6, 38^, or was even absent^39^. To shed more light on the possible pathophysiological role of FGF23 in the development of hypertrophy and heart failure we employed TAC, a pressure overload-induced hypertrophy model. We demonstrated that serum levels of intact Fgf23 (iFgf23) rose dramatically after pressure overload. Serum levels of iFGF23 are regulated both on the transcriptional level and by posttranslational regulation of FGF23 cleavage. Furin and furin-like proteases cleave iFGF23 into inactive fragments^40^, a process which is inhibited by GalNT3-mediated O-glycosylation at the cleavage site. Conversely, phosphorylation of FGF23 near the cleavage site by Fam20c prevents O-glycosylation, and facilitates furin-mediated cleavage^30^. Here, we report the novel finding of pressure overload-induced upregulation of *GalNT3* gene expression in the heart. This finding may not only be relevant for increasing local levels of iFgf23, but also for posttranslational modification and function of other proteins.

Pressure overload enhanced *Fgf23* gene transcription in heart and bone in our study. So far, several factors have been shown to regulate expression of the *Fgf23* gene such as phosphate^41^, 1,25(OH)_2_D_3_^42^, parathyroid hormone^43^, inflammation^11^, hypoxia, and iron deficiency^44^. However, it remains an open question why bone and heart respond to local cardiac stress with an increase in Fgf23 transcription. While the cardiac stimulus is still unknown, our study has identified aldosterone as an important stimulator of bony Fgf23 transcription after TAC, because mineralocorticoid receptor blockade with spironolactone normalised serum iFGF23 levels and bone *Fgf23* gene expression after TAC. In agreement with our finding, serum aldosterone levels correlated with circulating FGF23 levels in patients with non-ischemic cardiac disease^45^, and aldosterone increased Fgf23 production *in vitro* and in animal models^32^. Notably, spironolactone did not have any effect on serum and mRNA levels of *Fgf23* in control mice, indicating that aldosterone is not involved in the regulation of basal Fgf23 production. In line with that, spironolactone treatment did not reduce high Fgf23 plasma concentrations in *Klotho* deficient mice^46^. Interestingly, we did not observe any effect of spironolactone on cardiac *Fgf23* mRNA expression. Therefore, it is conceivable that different, tissue specific mechanisms are involved in the regulation of cardiac and bony *Fgf23* expression.

The physiological role of Klotho signalling in the heart is still controversial. Previous reports have shown that Klotho deficiency results in elevated iFgf23 levels^15^ which may *per se* induce LVH in a Klotho-independent fashion^47^, that soluble Klotho is protective in uremic and isoproterenol-induced cardiomyopathy^21, 48^, and that Klotho deficiency aggravated the stress-induced hypertrophic response due to dysregulation of cardiac TRPC6 channels^21, 48^. However, our study did not show any evidence of a negative effect of Klotho deficiency on cardiac phenotype and on development of LVH during pressure overload. One of the possible explanations for this discrepancy is that Klotho^−/−^/VDR^Δ/Δ^ mice on rescue diet used in the current study displayed no mineral disturbance, whereas others used Klotho^−/−^ mice, characterised by 1,25(OH)_2_D_3_-mediated hypercalcemia and hyperphosphatemia.

In our study we provided evidence that Fgf23 is not essentially mediating pressure-induced cardiac hypertrophy in a TAC model, using two independent approaches: i) by spironolactone-induced Fgf23 suppression in WT mice, and ii) by genetic deletion of Fgf23 in *Fgf23* ^−/−^/VDR^Δ/Δ^ mice. In both approaches, TAC-induced LVH developed independently of the presence or absence of Fgf23, and was not modulated by high or normal circulating iFgf23 levels. In light of the solid evidence from clinical and experimental studies showing a strong association between circulating iFGF23 and LVH in CKD, this is a perplexing finding. However, there are several explanatory scenarios for this apparent discrepancy. First, the specific type of cardiac load influences the type of cardiac hypertrophy in CKD and during pressure-overload^49^. CKD is a condition where volume overload is a major contributor of cardiovascular complications^50^. Fluid retention predicted risk of cardiovascular morbidity and mortality in CKD patients better than hypertension^50^. Differential signalling pathways are activated during volume overload-induced LVH compared to pressure overload-induced LVH^51^, suggesting involvement of different pro-hypertrophic mediators. Secondly, the most compelling mechanistic explanation of FGF23’s involvement in CKD-related hypertrophy is based on studies where FGFR4 but not FGF23 were selectively targeted by monoclonal antibodies^25^. Therefore, it remains unknown whether signalling molecules other than FGF23 may signal through FGFR4 in order to induce LVH in CKD. An essential role of paracrine FGF2 has been demonstrated in pressure overload-induced cardiac hypertrophy, as genetic deletion of *Fgf2* protected from hypertrophic changes^33^. In the current study, we confirmed increased cardiac *Fgf2* transcription in TAC mice. Thus, we suggest that increased FGF23 levels are not necessarily essential for the hypertrophic response in the presence of other molecules which may have a competing affinity for the same receptor, leading to the signalling switch associated with LVH. Changes in the pattern of FGF receptor expression may facilitate such a switch in cardiac tissue during pressure overload. Unlike in CKD, where gene expression of FGFR4 is stimulated in the myocardium^35^, we found increased *Fgfr1* and *Fgfr3*, but unchanged *Fgfr4* cardiac mRNA expression in our TAC pressure overload model. Others also reported that Fgfr1 and Fgfr3 are the most abundant FGFRs in the heart^52^. Further studies will be necessary to disentangle the differences in FGFR signalling between the different cardiac hypertrophy models.

A limitation of the current study is that the *Fgf23* and *Klotho* deficient animal models were on a VDR deficient genetic background to avoid early lethality. In addition, it is necessary to feed the VDR^Δ/Δ^ mice a chow enriched in phosphate and calcium (rescue diet) in order to avoid hypocalcaemia in these mice. However, our data indicate that absence of a functioning VDR does not blunt the pro-hypertrophic actions of FGF23 *in vivo*, and does not interfere with the TAC-induced increase in circulating Fgf23 or with the TAC-induced cardiac hypertrophy. Nevertheless, we cannot completely rule out an interaction between vitamin D and Fgf23 signalling in the heart.

Collectively, the results of the current study provide novel insights into the regulation and the role of Fgf23 in pressure overload-induced cardiac hypertrophy. Although we found a profound upregulation of circulating iFgf23 as well as of cardiac and bony Fgf23 transcription after TAC, our data do not support an essential role of Fgf23 or Klotho in the pathophysiology of pressure overload-induced cardiac hypertrophy. Thus, the pathophysiological role of elevated Fgf23 levels during pressure overload remains to be clarified.

## Methods

### Animals

All animal procedures were undertaken in accordance with European guidelines for animal experiments (EU RL 2010/63/EU) and approved by the Ethical Committees of the University of Veterinary Medicine Vienna and of the Austrian Federal Ministry of Science, Research and Economy. Adult male WT, VDR^Δ/Δ^, *Fgf23* ^−/−^/VDR^Δ/Δ^, and *Klotho* ^−/−^/VDR^Δ/Δ^ mice were bred and genotyped as previously described^27, 28^. Mice were maintained on rescue diet (Sniff™) enriched in calcium (2.0%), phosphorous (1.25%) and lactose (20%) in order to normalize mineral homeostasis in VDR-ablated mice^53^. All animals were kept in groups of 2–7 mice at 22–24 °C and a 12 h light/12 h dark cycle with free access to tap water and food.

A group of male 3-month-old WT and VDR^Δ/Δ^ mutant mice (n = 3–4 per group) received daily intraperitoneal injections of vehicle (phosphate-buffered saline with 2% DMSO) or 10 µg recombinant human FGF23 R176/179Q (rFGF23, kindly provided by Dr. Moosa Mohammadi, New York University School of Medicine) per mouse.

### Transverse aortic constriction

Transverse aortic constriction (TAC) or sham surgeries were performed in 3–4-month-old male mice under general anaesthesia induced with ketamine/medetomidine (100/0.25 mg/kg i.p.). Animals were endotracheally intubated, and ventilated with a tidal volume of 200 μL and a frequency of 200 breathing cycles per min using a small animal ventilator (SAR-1000; CWE Incorporated). After sternotomy, a ligation was placed between the origins of the brachiocephalic and left common carotid arteries around a 24- or 27-gauge needle, using a 6–0 silk suture, followed by prompt removal of the needle. Because preliminary experiments demonstrated more robust cardiac hypertrophy development using ligation with a 27-gauge needle, all remaining experiments in the study were performed accordingly. Sham animals underwent the same procedure without the aortic ligation. Analgesic (buprenorphine 0.25 mg/kg s.c.) and antibiotic (enrofloxacin, 10 mg/kg s.c) were injected for 4 and 5 days, respectively. Animals were killed 2 or 4 weeks after surgery by exsanguination from the abdominal vena cava under ketamine/xylazine anaesthesia (70/7 mg/kg i.p.). Tissue and serum samples were harvested, flash frozen and stored at −80 °C, or processed for histological analysis.

### Spironolactone treatment

Some mice were randomized to treatment with spironolactone (20 mg/kg in 0.7% ethanol) or vehicle (0.7% ethanol) administered in 100 µl by daily gavage. Treatment started one day before TAC or sham surgery and was continued until the end of the study, 2 weeks post-surgery.

### Transthoracic Doppler echocardiography

Echocardiography was performed 2 and 4 weeks after surgery using a 14 MHz linear transducer (Siemens Accuson s2000) under 1% isoflurane anaesthesia. Left ventricular (LV) wall thickness, internal dimensions and fractional shortening were evaluated in anatomic M-mode recorded in the short axis view at the papillary muscles level. Diastolic flow through the mitral valve was measured to evaluate LV diastolic function using pulsed-wave Doppler in the apical 4-chamber view. Success of the aortic constriction was evaluated from flow velocities measured in the thoracic aorta distal to the ligation. At least 5 cardiac cycles were averaged for each measured parameter.

### Central arterial and cardiac pressure measurement

Aortic and cardiac pressures were assessed using a SPR-671NR pressure catheter (1.4 F, Millar Instruments, Houston, TX, USA). Central arterial pressure was measured by inserting the catheter into the ascending aorta via the carotid artery under 1.0% isoflurane anaesthesia. The catheter was then advanced into the LV for measurement of cardiac pressure parameters. Pressure traces were recorded over 5 min, and analysed using LabChart7 software.

### Serum and urine biochemistry

Serum and urinary sodium, potassium, phosphorus, calcium, iron, and creatinine were analysed using a Cobas c111 analyser (Roche). Serum aldosterone (NovaTec), intact FGF23 (Kainos), and C-terminal Fgf23 (Immutopics) were assessed by commercially available ELISAs.

### RNA isolation and quantitative RT-PCR

Total RNA was isolated from flash-frozen tissue after homogenization using TRI Reagent® Solution (Invitrogen). The concentration and purity of isolated RNA were determined spectrophotometrically (NanoDrop 2000; ThermoScientific). 1 µg of RNA was reverse transcribed (High Capacity cDNA Reverse Transcription Kit; Applied Biosciences). Quantitative RT-PCR was performed on a Vii7 device (Applied Biosystems®) using the 5x Hot Firepol® Eva Green kit (Solis Biodyne). To exclude amplification of genomic DNA, primers were designed as exon spanning and their sequence is available upon request. A product melting curve analysis was performed to exclude primer dimerization and nonspecific amplification. All samples were measured in duplicate and expression values were normalized to ornithine decarboxylase antizyme 1 (*Oaz1*) mRNA.

### Histological evaluation of cardiac hypertrophy and fibrosis

Hearts were fixed in 4% paraformaldehyde for 24 h, embedded in paraffin and sectioned at a thickness of 5 µm. Fibrotic tissue was visualised by staining with picrosirius red according to a standard protocol. Collagen content was evaluated using ImageJ from the whole circumference of the left ventricle and septum at the level of the papillary muscles, and expressed as ratio of collagen-stained area to total muscle area of the left ventricle and septum. For the analysis of cardiomyocyte size, cardiac sections were stained with FITC-labelled wheat germ agglutinin. Cardiomyocyte size was evaluated using a semi-automated procedure by ImageJ in at least 800 cardiomyocytes from various regions of LV and septum. Fibrosis and cardiomyocyte size were each evaluated by 2 independent investigators in a blinded manner.

### Immunohistochemistry

Antigen retrieval was performed by heating the de-paraffinised cardiac sections to 100 °C for 15 min in citrate buffer (pH 6). Sections were then treated for 30 min with blocking solution containing 10% goat serum and 0.02% Triton X in PBS to prevent unspecific antibody binding. Primary antibody anti-phospho-NFATc4 (rabbit polyclonal IgG, 1:100 in blocking solution, St John’s Laboratory) were incubated for 60 min at room temperature. After washing, secondary biotinylated antibody (1:500, Vector) was added for 60 min at room temperature. Signal was developed by incubation with streptavidin-peroxidase (KPL-HistoMark) followed by 3-amino-9-ethyl carbazol (AEC, Invitrogen) staining. Sections were counter-stained with haematoxylin.

### Western blotting

Protein lysates from LV tissue were prepared using RIPA buffer according to standard protocols. Primary antibodies against FGF receptor 1 (FGFR1) (rabbit polyclonal IgG, D8E4, Cell Signalling) and GAPDH (Millipore) were used. After washing, membranes were incubated with horseradish peroxidase-conjugated secondary antibodies (Amersham Life Sciences), and bound antibody was detected using ECL reagent (Bio Rad). Quantification of Western blots was performed using ImageJ software. Protein expression was normalized to the expression of GAPDH.

### Data Availability

All data generated or analysed during this study are included in this published article (and its Supplementary Information files).

### Statistical analysis

Data are presented as mean ± SEM. Statistical analysis was performed using GraphPad Prism 6 and statistical software R version 3.32 (R Development Core Team, 2016). Comparisons between two groups were performed by two-sided t-test. Three- or more group comparisons within one genotype (WT) were performed using one-way ANOVA. In order to test for genotype-specific differences in TAC *vs*. sham surgery effects, two-way ANOVA models were used with the investigated parameter as dependent variable and genotype and surgery group as independent variables. For each genotype (VDR^Δ/Δ^, *Fgf23* ^−/−^/VDR^Δ/Δ^ and *Klotho* ^−/−^/VDR^Δ/Δ^) the mean parameter values in the TAC and SHAM surgery group and a 95% confidence interval for the difference of means were derived. Separate models were specified to compare genotypes, VDR^Δ/Δ^ vs *Fgf23* ^−/−^/VDR^Δ/Δ^ and VDR^Δ/Δ^ vs *Klotho* ^−/−^/VDR^Δ/Δ^. A potential genotype-specific difference in the surgery effect was assessed by testing for a significant interaction between genotype and surgery within the ANOVA model. Normality assumptions for confidence intervals and ANOVA residuals were assessed visually. All tests were two-sided and p values less than 0.05 were considered statistically significant.

## Electronic supplementary material

Supplementary Information
