# Supplementary material for: Genetic Ablation of Fgf23 or Klotho Does not Modulate Experimental Heart Hypertrophy Induced by Pressure Overload
Source: Sci Rep. 2017 Sep 12;7:11298. doi: 10.1038/s41598-017-10140-4 (PMC5595838; doi:10.1038/s41598-017-10140-4)

Supplementary material

Genetic Ablation of *Fgf23* or *Klotho* Does not Modulate Experimental Heart Hypertrophy Induced by Pressure Overload

Svetlana Slavic[1], Kristopher Ford[1], Magalie Modert[1], Amarela Becirovic[1], Stephan Handschuh[2], Andreas Baierl[3], Nejla Katica[1], Ute Zeitz[1], Reinhold G. Erben[1], [*]Olena Andrukhova[1]

[1] Department of Biomedical Sciences, University of Veterinary Medicine Vienna, Vienna, Austria

[2] VetCore, University of Veterinary Medicine Vienna, Vienna, Austria

[3] Department of Statistics and Operations Research, The University of Vienna

[*]**Corresponding author**:

Olena Andrukhova, PhD

Institute of Physiology, Pathophysiology, and Biophysics

Dept. of Biomedical Sciences,

University of Veterinary Medicine, Veterinaerplatz 1, 1210 Vienna, Austria

Phone +43-1-250 77 4560, Fax +43-1-250 77 4599

E-mail Olena.Andrukhova@vetmeduni.ac.at

Supplemental Figure 1: Characterisation of the transverse aortic constriction (TAC) mouse model

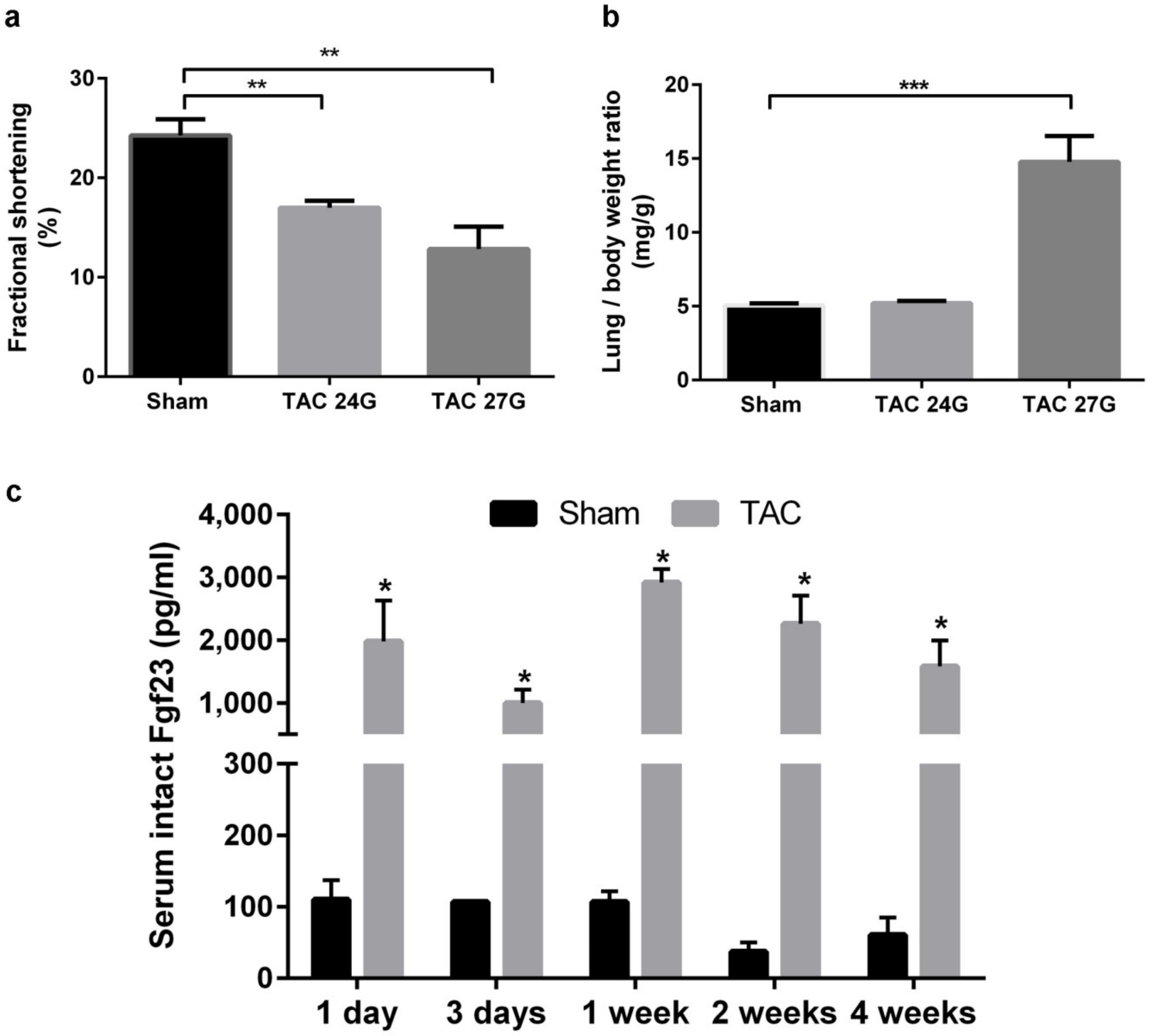

(a) Reduced cardiac function after TAC as measured by fractional shortening using echocardiography, 4-weeks post-surgery (n=6-7) (b) lung/body weight ratio are significantly increased after TAC when constriction was performed with a 27G-needle (n=6-7). (c). Time course of serum intact Fgf23 levels measured on day 1 and 3, as well as at 1, 2, and 4-weeks post-surgery (n=3-5). Data are mean ± SEM, *p<0.05, **p<0.01, ***p<0.001 vs. sham.

Supplemental Figure 2: Effect of spironolactone treatment on urinary Na^+ and K^+ excretion, GFR, cardiac hypertrophy and lung oedema 2-weeks after TAC

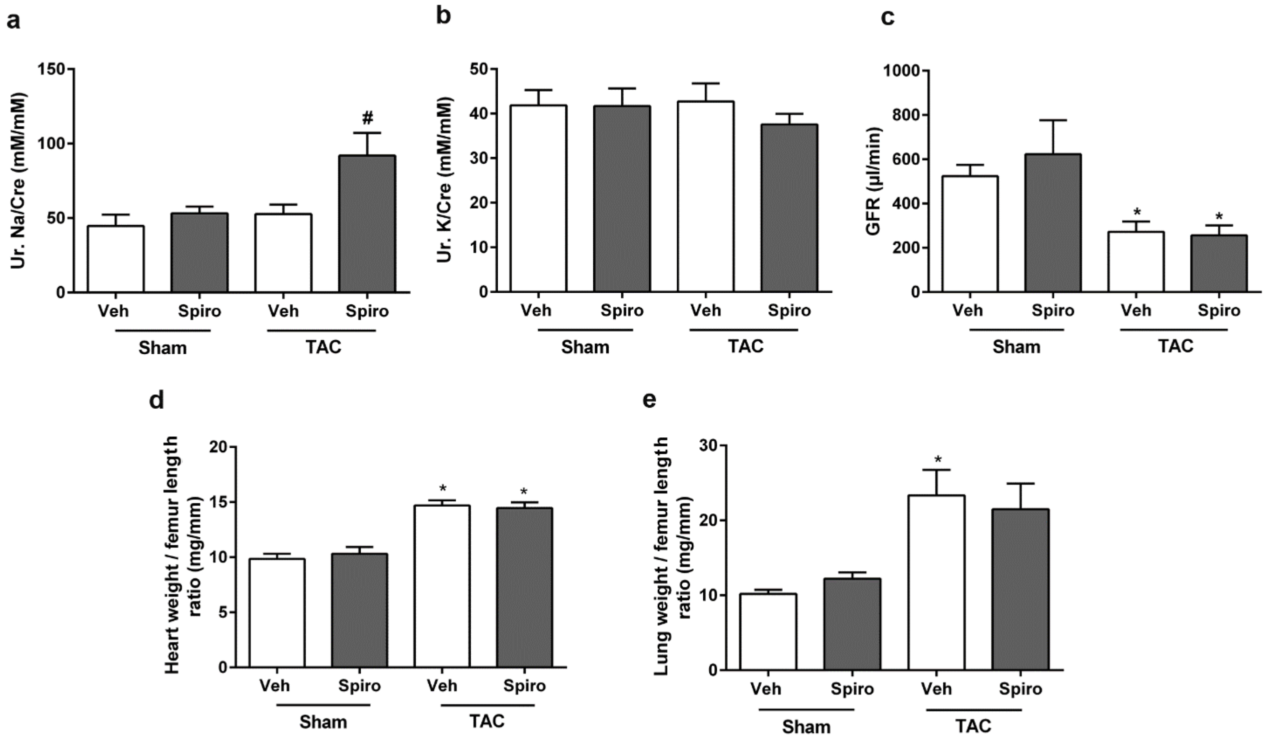

(a) Urinary Na^+ (b) K^+ excretion and (c) Glomerular filtration rate (GFR) in vehicle (Veh) or spironolactone (Spiro) treated wt animals 2-weeks after Sham or TAC surgery. (d) Heart weight and (e) lung weight were normalised to the femur length. Data are mean ± SEM, n=4-7, #p<0.05 vs. TAC Veh, *p<0.05 vs. respective sham control.

Supplemental Figure 3: VDR deficiency does not alter the afterload- and rFGF23-induced cardiac hypertrophy

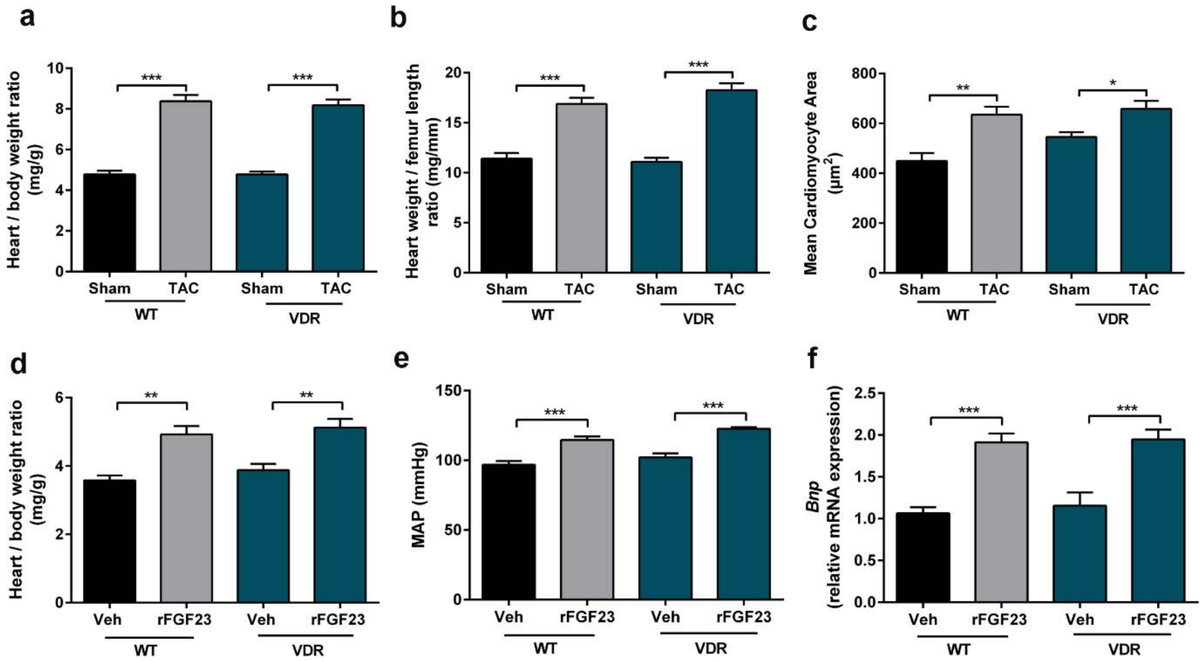

(a) Heart weight normalised to body weight in 4-weeks post-surgery (n=6-9). (b) Heart weight normalised to femur length 4-weeks post-surgery (n=6-9). (c) Mean cardiomyocyte area after FITC-WGA staining 4-weeks post-surgery (n=6-9). (d) Heart weight to body weight ratio after 5-days of i.p. injections with recombinant FGF23 (rFGF23) (n=4-5). (e) Mean arterial pressure measured by intra-aortic pressure catheter after 5-days of rFGF23 i.p. injections (n=4-5). (f) Cardiac mRNA expression of brain natriuretic peptide (Bnp) after 5-days of rFGF23 i.v. injections (n=3). Data are mean ± SEM, *p<0.05, **p<0.01, ***p<0.001.

Supplemental Figure 4: Genetic deletion of *Fgf23* or *Klotho* does not affect survival and left ventricular geometry in afterload-induced cardiac hypertrophy, 4-weeks after TAC surgery

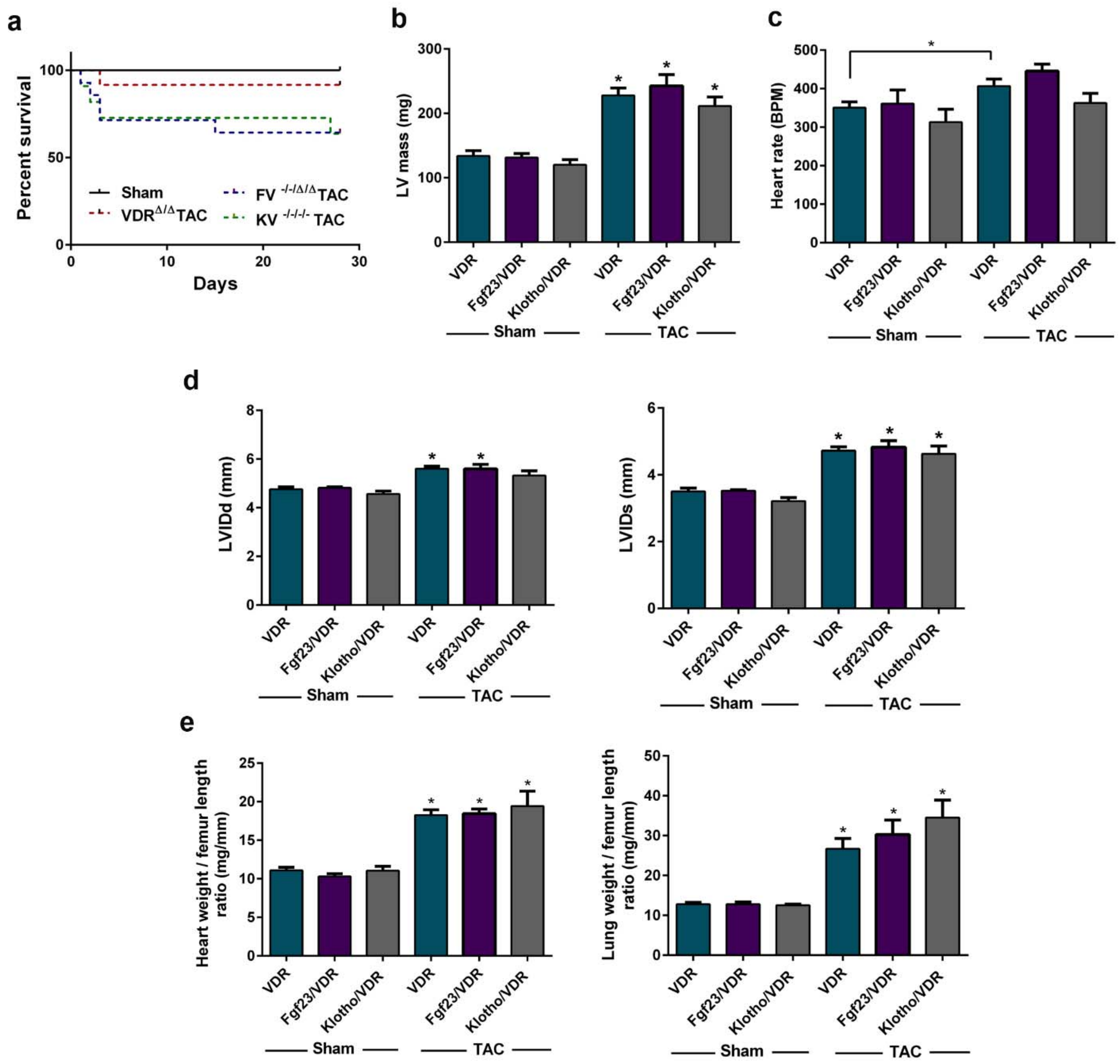

(a) Kaplan-Meier survival curves. All Sham-operated animals survived the surgery. Survival was not significantly reduced in Fgf23$^{-/-}$/VDR$^{\Delta/\Delta}$ and Klotho$^{-/-}$/VDR$^{\Delta/\Delta}$ mice compared to VDR$^{\Delta/\Delta}$ mice after TAC (n=6-11). (b) Left ventricular (LV) mass (n=6-9) evaluated by echocardiography. (c) Heart rate evaluated by intra-aortic pressure catheter (n=6-9) (d) LV internal diameter in diastole (left) and LV internal diameter in systole evaluated from echocardiography (n=6-9). (e) Heart weight (left) and lung weight (right) normalised to femur length (n=6-9). Data are mean ± SEM. *p<0.05 vs. respective sham control.

Supplemental Figure 5: Cardiac *Fgf2* mRNA expression and ERK1/2 activation in afterload-induced cardiac hypertrophy

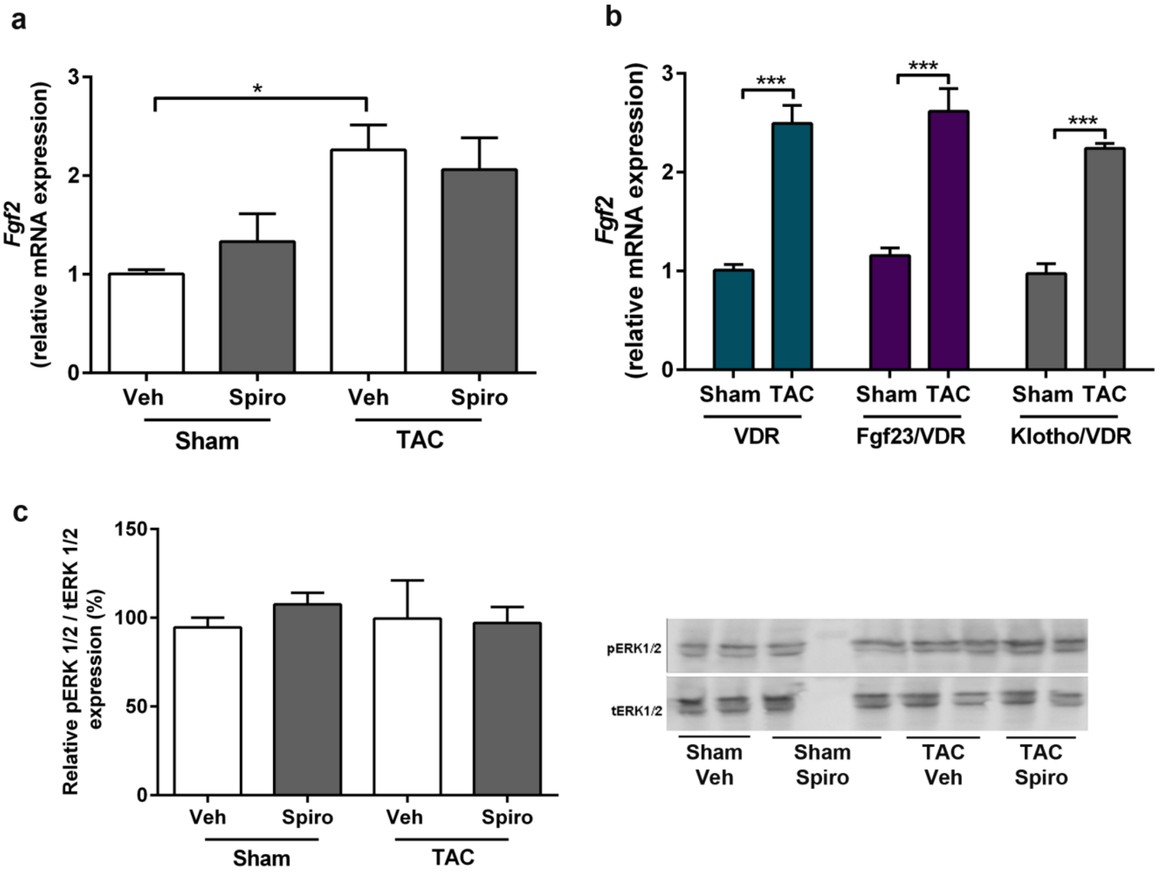

(a) Left ventricle mRNA expression of *Fgf2* in vehicle (Veh) or spironolactone (Spiro) treated wt animals 2-weeks after Sham or TAC surgery (n=3-7). (b) Left ventricle mRNA expression of *Fgf2* in $VDR^{\Delta/\Delta}$, $Fgf23^{-/-}/VDR^{\Delta/\Delta}$ and $Klotho^{-/-}/VDR^{\Delta/\Delta}$ mice 4-weeks after sham and TAC surgery (n=4-6). (c) Western blot analysis of pERK1/2 expression normalised to the total ERK1/2 abundance in left ventricle homogenate (n=2 per group). Data are mean ± SEM, *p<0.05 and ***p<0.001.

Supplemental Figure 6: Effect of spironolactone, Fgf23 and Klotho deficiency on cardiac Calcineurin-NFAT signalling

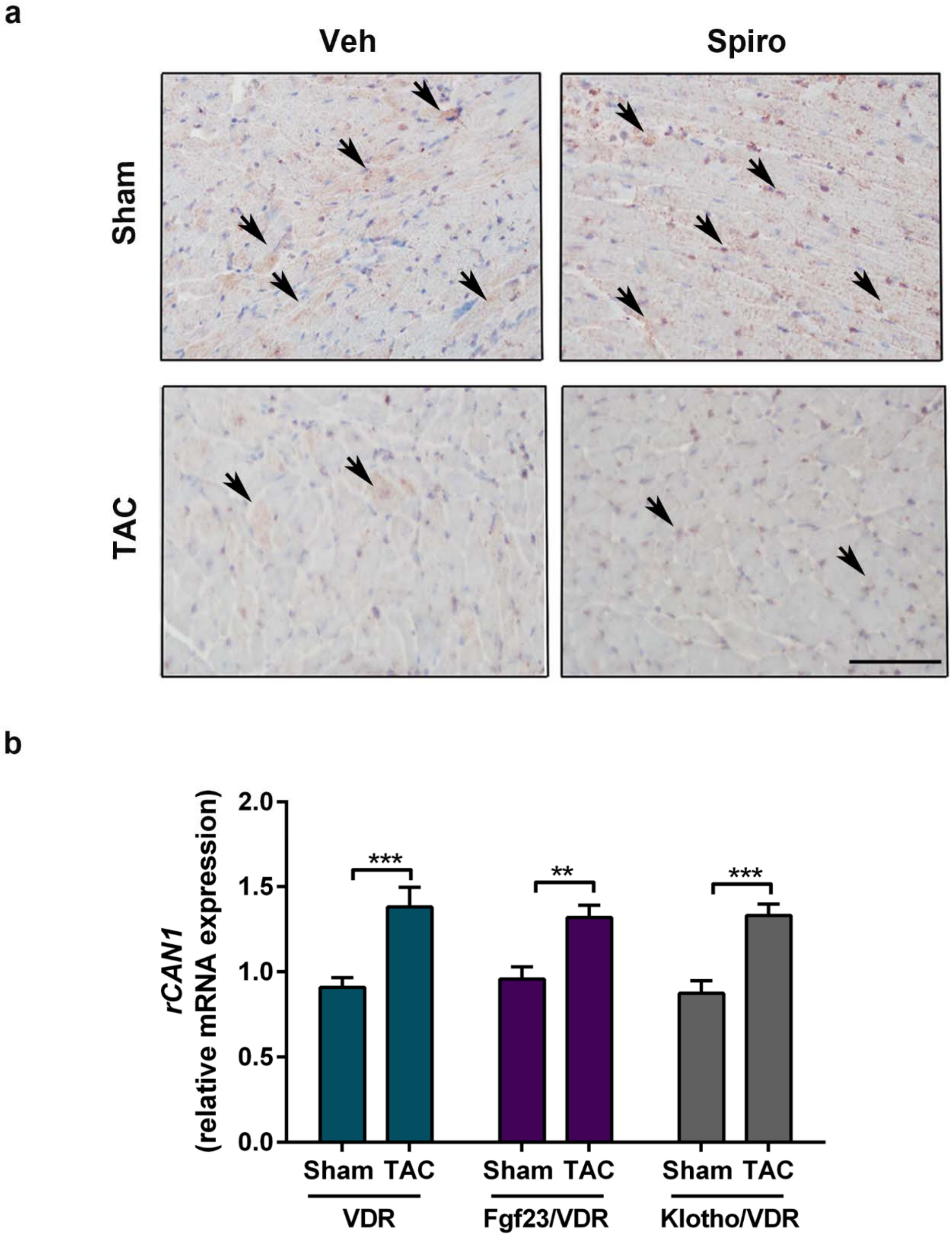

(a) Representative images of immunohistochemical anti-pNFAT staining (arrows indicate positive staining in red colour) of cardiac sections in wt mice 2-weeks after Sham or TAC surgery. Scale bar represents 100µm. (b) Left ventricle mRNA expression of *rCAN1* in $VDR^{\Delta/\Delta}$, *Fgf23*$^{-/-}$/$VDR^{\Delta/\Delta}$ and *Klotho*$^{-/-}$/$VDR^{\Delta/\Delta}$ mice 4-weeks after sham or TAC surgery (n=5-6). Data are mean ± SEM, $^{**}p<0.01$ $^{***}p<0.001$.

Supplemental Figure 7: Fgf receptor expression in the left ventricle 4-weeks after TAC surgery

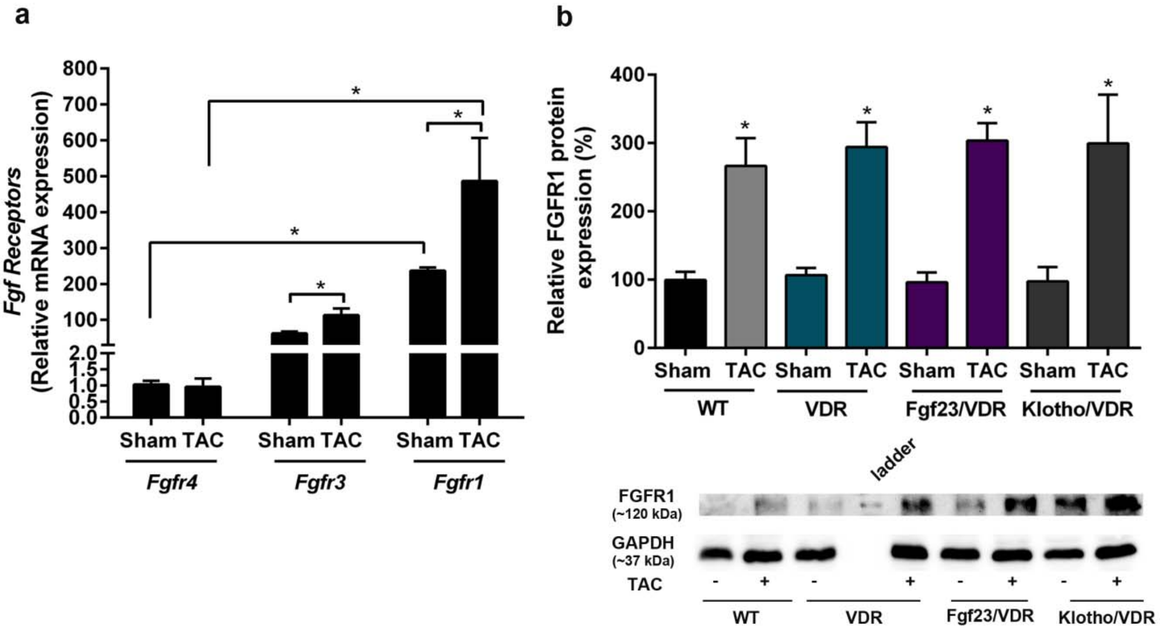

(a) Left ventricle mRNA expression of *Fgfr1*, *Fgfr3* and *Fgf4* in wild-type mice 4-weeks after Sham or TAC surgery, normalised to Sham values of *Fgf4* expression (n= 6 per group). (b) Western blot analysis of FGFR1 protein expression in the left ventricle of wt, VDR$^{\Delta/\Delta}$, *Fgf23*$^{-/-}$/VDR$^{\Delta/\Delta}$ and *Klotho*$^{-/-}$/VDR$^{\Delta/\Delta}$ mice 4-weeks after sham or TAC surgery (n=3 per group). Data are mean ± SEM, in (a) *p<0.05, in (b) *p<0.05 vs. respective sham control.

Supplemental Table 1. Serum biochemistry in WT mice 4-weeks after Sham or TAC surgery

	Sham	TAC	p Value
**Alkaline Phosphatase (U/L)**	63,1 ± 3,7	76,6 ± 4,9	**p<0,05**
**Albumin (g/L)**	30,2 ± 1,1	29,5 ± 0,9	n.s.
**Urea (mmol/L)**	7,87 ± 0,6	9,80 ± 0,4	**p<0,05**
**Creatinine (µmol/L)**	8,46 ± 0,5	11,5 ± 0,6	**p<0,01**
**Phosphate (mmol/L)**	2,81 ± 0,3	3,38 ± 0,3	n.s.
**Calcium (mmol/L)**	2,31 ± 0,1	2,42 ± 0,1	n.s.
**Sodium (mmol/L)**	149,6 ± 1,0	152,3 ± 1,5	n.s.
**Potassium (mmol/L)**	4,17 ± 0,3	4,38 ± 0,3	n.s.
**Iron (nmol/L)**	26,1 ± 2,4	27,8 ± 1,1	n.s.

Data are mean ± SEM, n=7 per group.

Suppl. Table 2. Central arterial and cardiac pressure measurement 4-weeks after Sham or TAC surgery.

	**$VDR^{\Delta/\Delta}$**		**$Fgf23^{-/-}/VDR^{\Delta/\Delta}$**		**$Klotho^{-/-}/ VDR^{\Delta/\Delta}$**	
	**Sham**	**TAC**	**Sham**	**TAC**	**Sham**	**TAC**
**Mean Pressure (mmHg)**	81,2±2,5	98,74±3,0*	79,3±3,5	98,2±3,6*	77,0±1,8	96,1±4,9*
**Max dP/dt (mmHg/s)**	8243±267	6670±513*	7370±648	6233±1220	7231±692	5565±428
**Contractility (1/s)**	162,6±6	108,3±6,8*	148,8±14,4	103,7±19,2	136,4±12	94,6±14,4
**Tau (ms)**	11,1±0,5	21,4±2,6*	14,0±1,4	20,6±4,5	15,2±1,7	22,7±3,7
**EDP (mmHg)**	7,3±2,7	29,04±2,9*	10,7±2,9	27,6±5,3*	7,40±1,6	24,9±5,1

Data are mean ± SEM, n=5-9 per group, *p<0.05 vs. sham control of the same genotype.

Uncropped gels for Figure 3b.

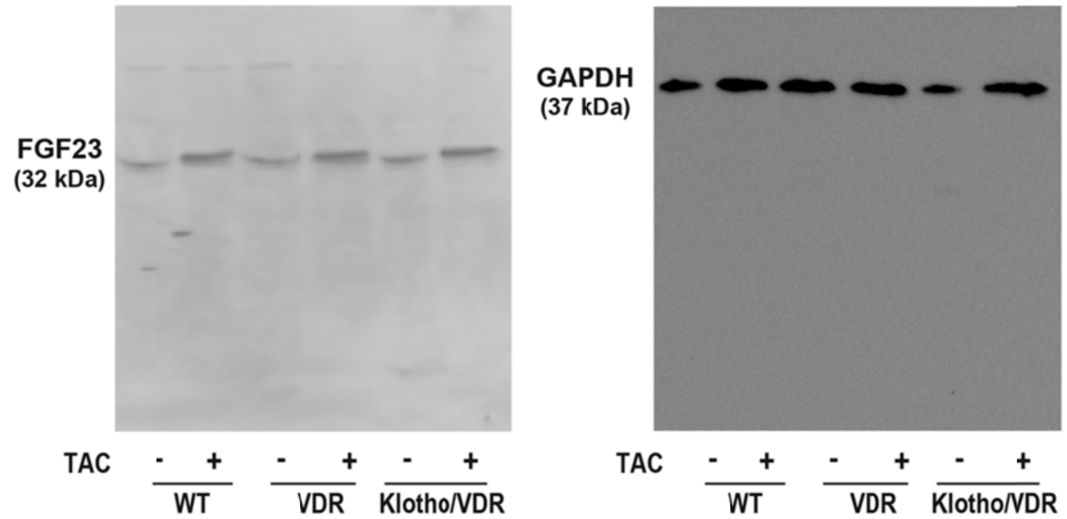

Supplement: Supplementary file 1 — Supplementary Information [file 41598_2017_10140_MOESM1_ESM.pdf]
